# *Borrelia miyamotoi* and *Candidatus* Neoehrlichia mikurensis in *Ixodes ricinus* Ticks, Romania

**DOI:** 10.3201/eid2203.150140

**Published:** 2016-03

**Authors:** Zsuzsa Kalmár, Hein Sprong, Andrei D. Mihalca, Călin M. Gherman, Mirabela O. Dumitrache, Elena C. Coipan, Manoj Fonville, Vasile Cozma

**Affiliations:** University of Agricultural Sciences and Veterinary Medicine Cluj-Napoca, Romania (Z. Kalmár, A.D. Mihalca, C.M. Gherman, M.O. Dumitrache, V. Cozma);; National Institute of Public Health and Environment, Bilthoven, the Netherlands (H. Sprong, E.C. Coipan, M. Fonville)

**Keywords:** Borrelia miyamotoi, Candidatus Neoehrlichia mikurensis, Anaplasma phagocytophilum, bacteria, Ixodes ricinus, ticks, vector-borne infections, tickborne infections, questing, circulation, Romania

**To the Editor:** *Ixodes* spp. ticks are vectors for human and animal pathogens. *Ix. ricinus* ticks are widely distributed, frequently reported to feed on humans, and the main vector for a large variety of tickborne pathogens (*1*). The effect of ticks and tickborne diseases on public health, animal health and welfare, and animal production appears to be an increasing global problem, which will lead to considerable economic costs (*2*).

*Borrelia miyamotoi* is a spirochete that belongs to the relapsing fever group and causes symptoms similar to those of other relapsing fever group pathogens and Lyme borreliosis, including erythema migrans−like skin lesions (*3*). The geographic distribution of *B. miyamotoi* is sporadic; it has been detected in *Ixodes* spp. ticks in many countries in Europe and in North America and Asia. In Russia, the United States, and recently in the Netherlands, *B. miyamotoi* was detected in humans and confirmed to cause disease (*4*,*5*). In Romania, pathogens that cause Lyme borreliosis and reptile-associated borreliae were identified in different tick populations (*6*,*7*). However, no information is available on the presence of relapsing fever group borreliae in this country.

*Candidatus* Neoehrlichia mikurensis and *Anaplasma phagocytophilum* are obligate, intracellular, tickborne pathogens of the family *Anaplasmataceae*; both are emerging zoonotic agents. *Candidatus* N. mikurensis causes monocytotropic ehrlichiosis in canids and humans and granulocytic anaplasmosis in humans and domestic animals (*8*). These 2 pathogens are found throughout Europe in *Ix. ricinus* ticks (*8*). *A. phagocytophilum* has been reported in questing *Ix. ricinus* ticks, dogs, wild boars, hedgehogs, and tortoises in Romania (*9*). Recently, *Candidatus* N. mikurensis was detected in an *Ix. ricinus* tick that had bitten a human in Romania (*10*). This recently discovered tickborne agent was shown to be a risk for disease in humans and has been detected in questing *Ix. ricinus* ticks throughout Europe and in animal tissue samples and human patients (*8*).

Relapsing fever spirochetes and potential public health risks associated with tickborne pathogens are a serious medical problem. Thus, we assessed the presence of *B. miyamotoi*, *A. phagocytophilum*, and *Candidatus* N. mikurensis in questing *Ix. ricinus* ticks in Romania.

Questing *Ix. ricinus* ticks were available from previous studies conducted by our research group. A random sampling approach was used as described (*7*). To detect potentially pathogenic bacteria, 468 questing *Ix. ricinus* ticks were collected from 4 regions from Romania, randomly selected, and analyzed.

Detection of pathogens was performed by using multiplex quantitative PCRs (qPCRs) specific for the *flaB* and *ospA* genes of *B. miyamotoi*, the *msp2* gene of *A. phagocytophilum*, and the g*roEL* gene of *Candidatus* N. mikurensis. We used IQ Multiplex Powermix (Bio-Rad, Carlsbad, CA, USA) and a final reaction volume of 20 μL (*8*). For detection of *A. phagocytophilum* and *Candidatus* N. mikurensis, we also performed multiplex qPCR as described (*8*). For detection of *B. miyamotoi*, a specific region of the *flab* gene was targeted by using multiplex qPCR according to a previous described protocol (*1*). For quality control of qPCRs, we included positive and negative controls. Sequences of qPCR products were analyzed and compared with sequences available in GenBank.

*B. miyamotoi* was detected in 7 ticks: 2 (1.59%) of 126 males, 2 (0.68%) of 296 females, and 3 (6.52%) of 46 nymphs. *A. phagocytophilum* was detected in 16 ticks: 1 (0.79%) of 126 males, 11 (3.72%) of 296 females, and 4 (8.70%) of 46 nymphs. *Candidatus* N. mikurensis was detected in 25 ticks: 5 (3.97%) of 126 males, 18 (6.08%) of 296 females, and 2 (4.35%) of 46 nymphs. Overall prevalences were 1.50% for *B. miyamotoi*, 3.42% for *A. phagocytophilum*, and 5.34% for *Candidatus* N. mikurensis. Prevalences of each pathogen in specific varied by locality (Table). No co-infections were detected.

**Table:** Prevalence of 3 bacterial species in questing *Ixodes ricinus* in 14 localities, Romania

Locality (county)	No. ticks tested (nymphs, males, females)	No. (%) ticks positive, by bacterial species		
		*Borrelia miyamotoi*	*Anaplasma phagocytophilum*	*Candidatus* Neoehrlichia mikurensis
Cugir (Alba)	19 (8, 4, 7)	0	2 (10.53)	1 (5.26)
Vladimirescu (Arad)	17 (0, 5, 12)	0	2 (11.76)	0
Bicaci (Bihor)	23 (12, 5, 6)	0	4 (17.4)	0
Bistrița (Bistriţa-Năsăud)	30 (0, 10, 20)	0	0	0
Poiana Mărului (Brașov)	66 (0,10, 56)	2 (3.03)	0	0
Vultureni (Cluj)	44 (3, 10, 31)	1 (2.27)	3 (6.82)	2 (4.55)
Micești (Cluj)	62 (0, 15, 47)	1 (1.62)	0	2 (3.23)
Reșița (Caraș-Severin)	21 (0, 10, 11)	0	0	0
Corund (Harghita)	59 (7, 17, 37)	0	1 (1.7)	4 (6.78)
Bistra (Maramureș)	26 (1, 10, 15)	0	0	0
Icland (Mureș)	37 (6, 5, 26)	2 (5.41)	4 (10.81)	8 (21.62)
Mediaș (Sibiu)	12 (2, 4, 6)	1 (8.33)	0	2 (16.67)
Rătești (Satu Mare)	22 (7, 10, 15)	0	0	1 (4.55)
Lugoj (Timiș)	30 (0, 11, 19)	0	0	5 (16.67)
Total	468 (46, 126, 296)	7 (1.5)	16 (3.42)	25 (5.34)

We analyzed *flab*, *msp2*, and *groEL* gene sequences obtained by qPCR. These sequences showed 99%–100% identities with gene sequences of *B. miyamotoi* (GenBank accession no. KJ847050), *A. phagocytophilum* (accession no. KP164415), and *Candidatus* N. mikurensis (accession no. FJ966365).

In Romania, the density of *Ix. ricinus* ticks is high and their host diversity is extensive (*7*). However, data for effects of tickborne pathogens on public health are scarce in this country. In this study, we detected *B. miyamotoi*, *A. phagocytophilum*, and *Candidatus* N. mikurensis in questing *Ix. ricinus* ticks in Romania, which confirms the emerging trend of these pathogens in Europe. Because of the scarcity of information on human infections with these pathogens in Romania, serologic and molecular investigations and their implementation are needed for diagnosis, which might help in assessing the effect of these pathogens on public health.
